# Subtype-specific response of retinal ganglion cells to optic nerve crush

**DOI:** 10.1038/s41420-018-0069-y

**Published:** 2018-06-28

**Authors:** S. Daniel, AF Clark, CM McDowell

**Affiliations:** North Texas Eye Research Institute, Department of Pharmacology and Neuroscience, University of North Texas Health Science Center, Fort Worth, Texas United States

**Keywords:** Retina, Cell death in the nervous system

## Abstract

Glaucoma is a neurodegenerative disease with retinal ganglion cell (RGC) loss, optic nerve degeneration and subsequent vision loss. There are about 30 different subtypes of RGCs whose response to glaucomatous injury is not well characterized. The purpose of this study was to evaluate the response of 4 RGC subtypes in a mouse model of optic nerve crush (ONC). In this study, we also evaluated the pattern of axonal degeneration in RGC subtypes after nerve injury. We found that out of the 4 subtypes, transient-Off α RGCs are the most susceptible to injury followed by On–Off direction selective RGCs (DSGC). Non-image forming RGCs are more resilient with ipRGCs exhibiting the most resistance of them all. In contrast, axons degenerate irrespective of their retinal soma after ONC injury. In conclusion, we show that RGCs have subtype specific cell death response to ONC injury and that RGC axons disintegrate in an autonomous fashion undergoing Wallerian degeneration. These discoveries can further direct us towards effective diagnostic and therapeutic approaches to treat optic neuropathies, such as glaucoma.

## Introduction

Glaucoma is a group of optic neuropathies with clinical manifestations including cupping of the optic disc, thinning and loss of the retinal nerve fiber layer, and characteristic visual field defects^1^. It is the leading cause of irreversible blindness worldwide^2^. The initial site of injury in glaucoma is believed to be at the optic nerve head. Glaucomatous changes to the optic nerve head damages the axons of retinal ganglion cells (RGC), which are the output neurons of the retina that carry visual signals to the retino-recipient regions of the brain^2^. In most cases vision loss does not occur until the disease has progressed considerably and therefore glaucoma goes undiagnosed until later stages. Current treatment options are limited to lowering intraocular pressure (IOP) and can only manage the disease^3–6^. There is no treatment available to halt glaucoma progression or reverse the damage done to the RGCs. Therefore, early disease detection and treatment aimed at neuroprotection and regeneration are an imminent need.

Most mammals have approximately 30 different subtypes of RGCs that differ in size, morphology, dendritic arborization, and electrophysiological functions^7–12^. Thus, in order to understand the pathophysiology of glaucoma, it is important to evaluate the response of these subtypes individually rather than studying them as a single entity. The many similarities between a human and mouse eye, and the availability of various genetic tools, have made it possible to study glaucoma and its phenotypes in mice^13, 14^. One such tool is the development of transgenic mice expressing GFP in individual subtypes of RGCs^15–17^. By subjecting these animals to glaucomatous insults, we can study the effect of this injury exclusively in each particular subtype and have a deeper understanding of susceptibility of these RGCs to glaucomatous insults.

We utilized the well-established optic nerve crush^18^ (ONC) model in mice to mimic glaucomatous optic nerve axonal injury. This is an acute injury model with characteristic progression as seen in glaucoma^19^. Transgenic animals labeling 3 distinct subtypes of RGCs, namely the transient Off-α RGC subtype with central projection to the superior colliculus and dorsal lateral geniculate nucleus^15^, On–Off-direction selective (posterior motion) RGC subtype with central projection to the superior colliculus, dorsal and ventral lateral geniculate nucleus as well as to the zona incerta^16^, and Cadherin 3 expressing RGC subtype with central projections to non-image forming centers^17^, were subjected to ONC injury. We also used melanopsin antibody to label intrinsically photosensitive RGC or ipRGCs (non-image forming central projections) post ONC^20,21^.

According to current studies, there are 3 types of α-RGCs. These RGCs are large and express similar markers including neurofilament, spp1, kcng4, etc.^22^, but differ in their physiological properties, dendritic arborization and stratification in the inner plexiform layer and have unique molecular signatures with each type amounting to approximately 1% of the total RGCs^7^. On–Off direction selective ganglion cells (DSGCs) respond to visual motion stimuli. There are 4 types of On–Off DSGCs depending on the direction of the moving object to which they respond. They all have similar dendritic stratification and express CART (cocaine and amphetamine-regulated transcript) but have different physiological and molecular signatures with each type amounting to approximately 2–3% of the total RGCs^7,23^. Some RGCs have ancillary functions other than relaying of visual cues to the brain such as pupillary light reflexes and circadian rhythm^24^. Cadherin expressing RGC subtype and ipRGs have non-visual functions and they amount to approximately 1% and approximately 4% of the total RGCs, respectively^17,25^.

In this study, we evaluated the pattern of cell death in each subtype of RGC described above over a time course of 14 days. We also determined the pattern of axonal degeneration in these subtypes. Our aim was to establish whether these subtypes are differentially susceptible to the injury and whether axons follow a similar trend of degeneration as their respective cell somas. These data provide a basis for future studies to exploit individual RGC subtype electrophysiological and cellular properties to develop novel diagnostic techniques for early detection, and also develop effective neuroprotective and neuro-regenerative strategies to stop and reverse the progression of optic neuropathies, including glaucoma.

## Results

### RGC subtype somas are differentially susceptible to ONC injury

**Fig. 1:**
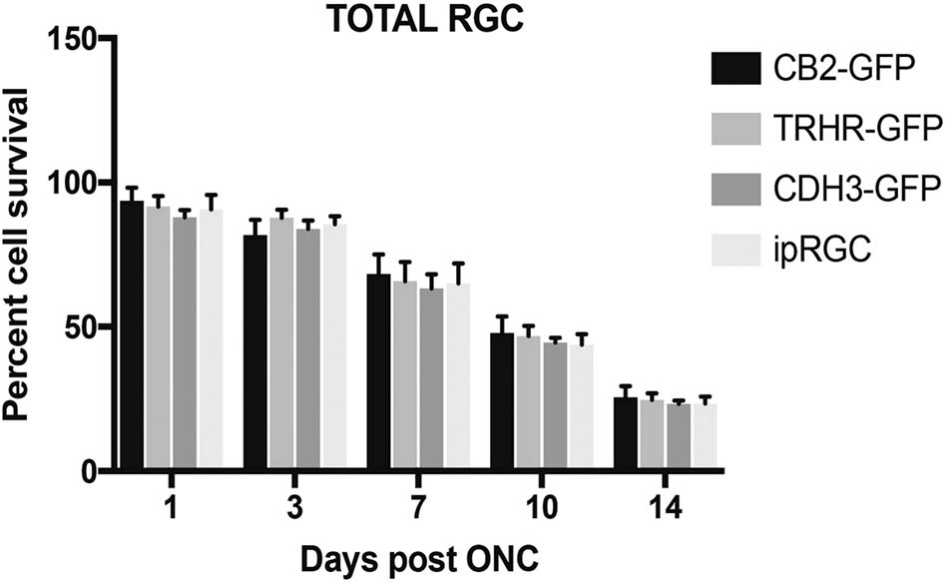
Comparison of total RGC loss after ONC. Percent cell survival of total RGCs between each strain within each time point shows no significant differences, by Two Way-ANOVA and Tukey’s post hoc test

**Fig. 2:**
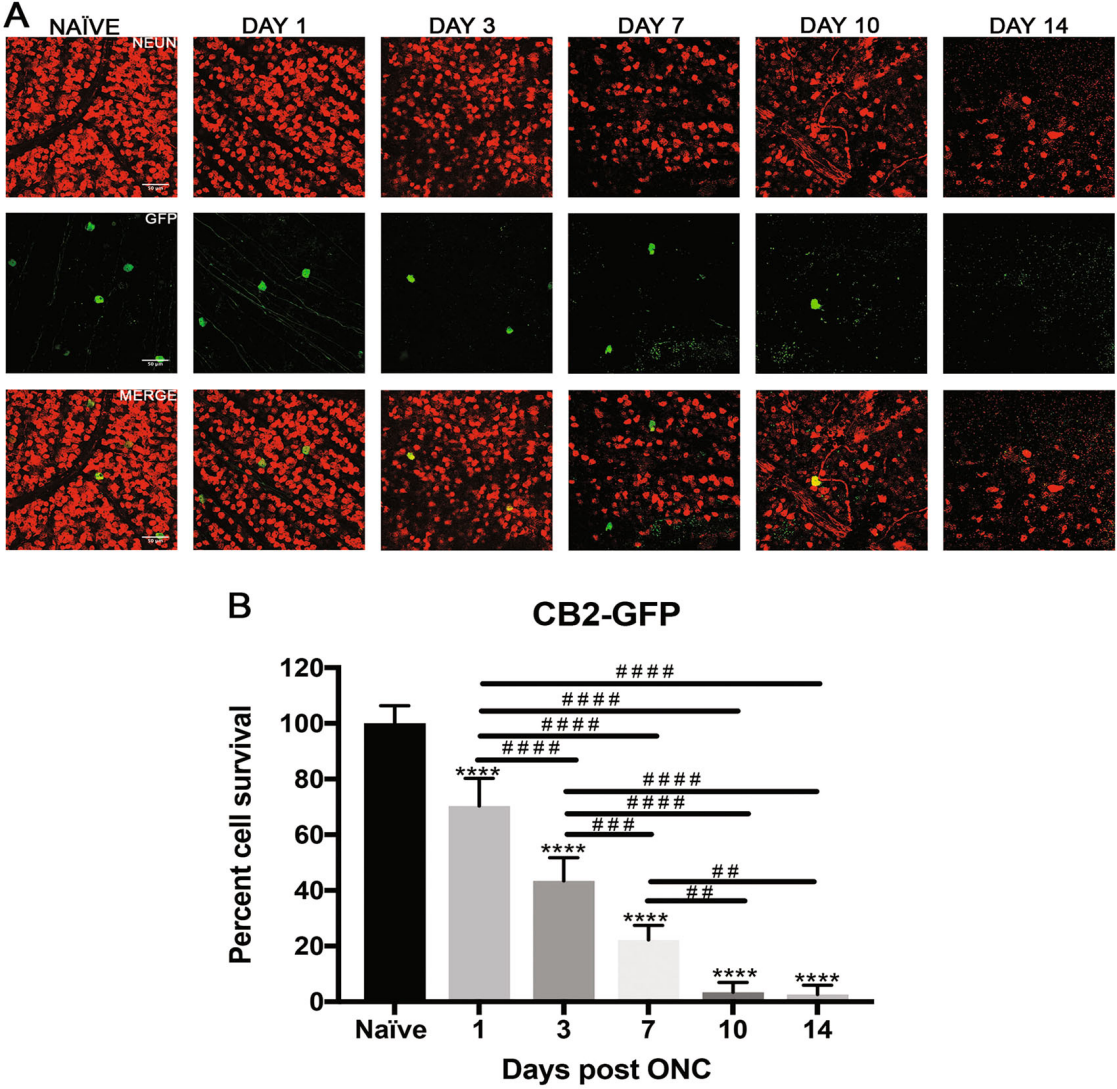
Effect of ONC injury on CB2-GFP RGCs. **a** Representative images showing NeuN (red) and GFP (green) immunolabeled retinal flat-mounts from transgenic CB2-GFP mice. Images of ONC (1 day, 3 days, 7 days, 10 days and 14 days post injury) and naïve control eyes are shown. Scale bar = 50 μm. **b** Percent cell survival of CB2-GFP RGCs. There was a steady decline in cell survival throughout the time course with significant cell loss at each time point when normalized to naïve. The values are represented as mean ± SD (*n* = 4–5). (* comparison to naïve, ^#^ comparison between time points). ****, ^# # # #^ *p* < 0.0001, ^# #^ *p* = 0.002, ^# # #^ *p* = 0.0005 by One Way-ANOVA and Tukey’s post hoc test

**Fig. 3:**
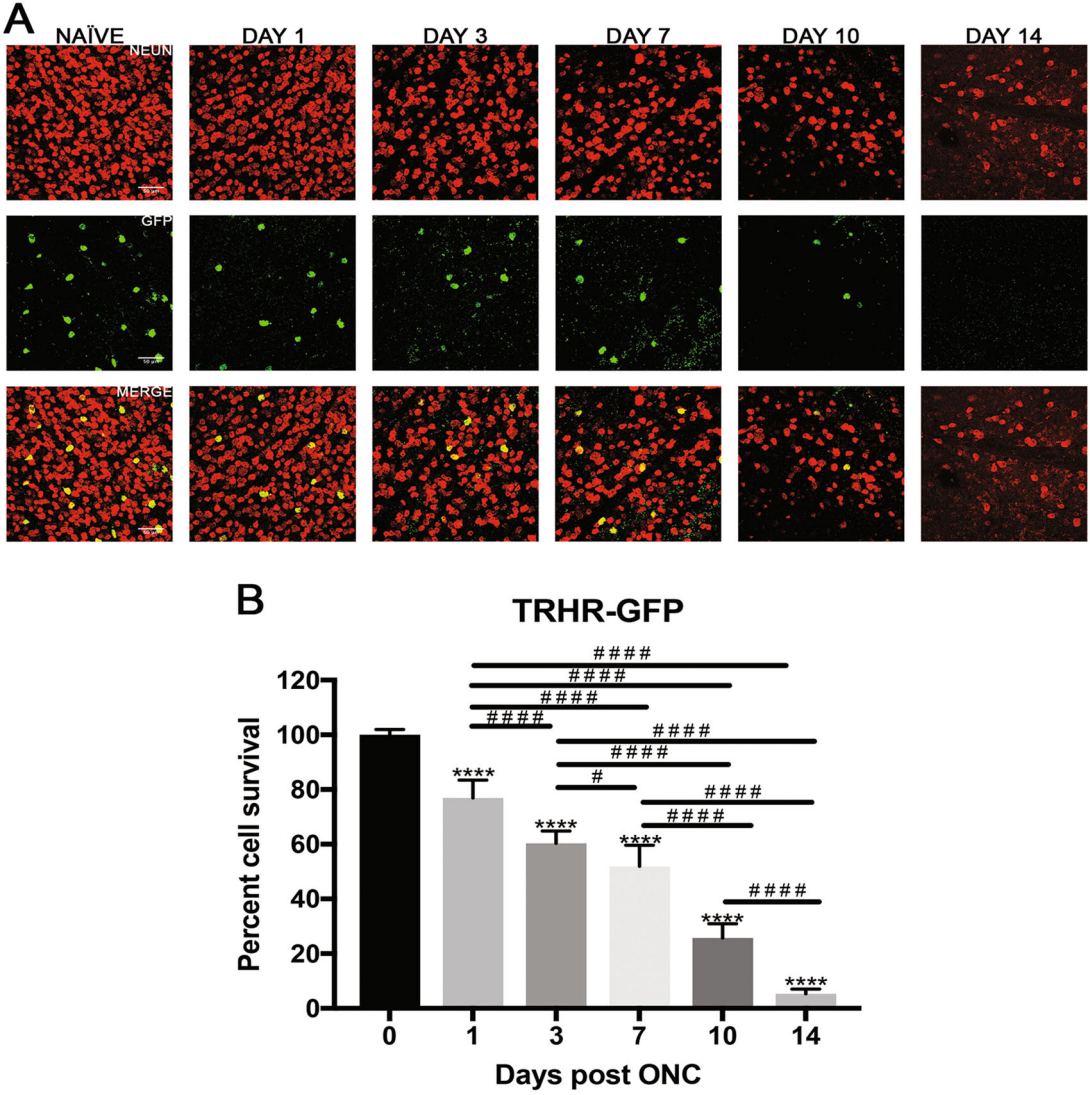
Effect of ONC injury on TRHR-GFP RGCs. **a** Representative images showing NeuN (red) and GFP (green) immunolabeled retinal flat-mounts from transgenic TRHR-GFP mice. Images of ONC (1 day, 3 days, 7 days, 10 days and 14 days post injury) and naïve control eyes are shown. Scale bar = 50 μm. **b** Percent cell survival of TRHR-GFP RGCs. There was significant decline in cell survival through the 14-day time course when normalized to naïve. The values are represented as mean ± SD (*n* = 7). (* Comparison to naïve, ^#^ comparison between time points). ^# # # #^, **** *p* < 0.0001, ^#^ *p* = 0.3 by One Way-ANOVA and Tukey’s post hoc test

**Fig. 4:**
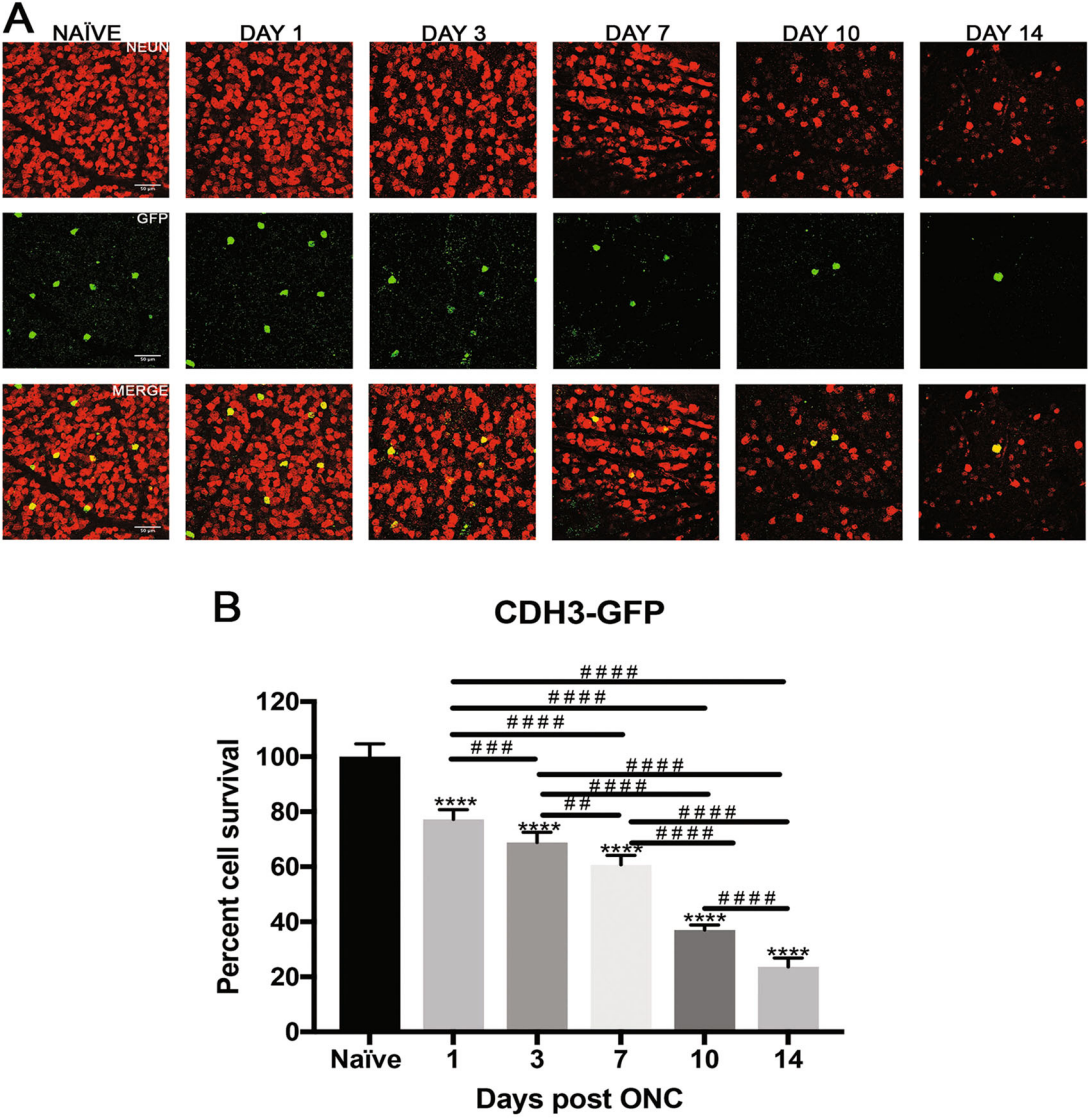
Effect of ONC injury on CDH3-GFP RGCs. **a** Representative images showing NeuN (red) and GFP (green) immunolabeled retinal flat-mounts from transgenic CDH3-GFP mice. Images of both ONC (1 day, 3 days, 7 days, 10 days and 14 days post injury) and naïve control eyes are shown. Scale bar = 50 μm. **b** Percent cell survival of CDH3-GFP RGCs. There was significant decline in cell survival through the 14-day time course when normalized to naïve. The values are represented as mean ± SD (*n* = 7). (* comparison to naïve, ^#^ comparison between time points). ****, 7^# # # #^ *p* < 0.0001, ***, ^# # #^ *p* = 0.0009, ^# #^ *p* = 0.001 by One Way-ANOVA and Tukey’s post hoc test

**Fig. 5:**
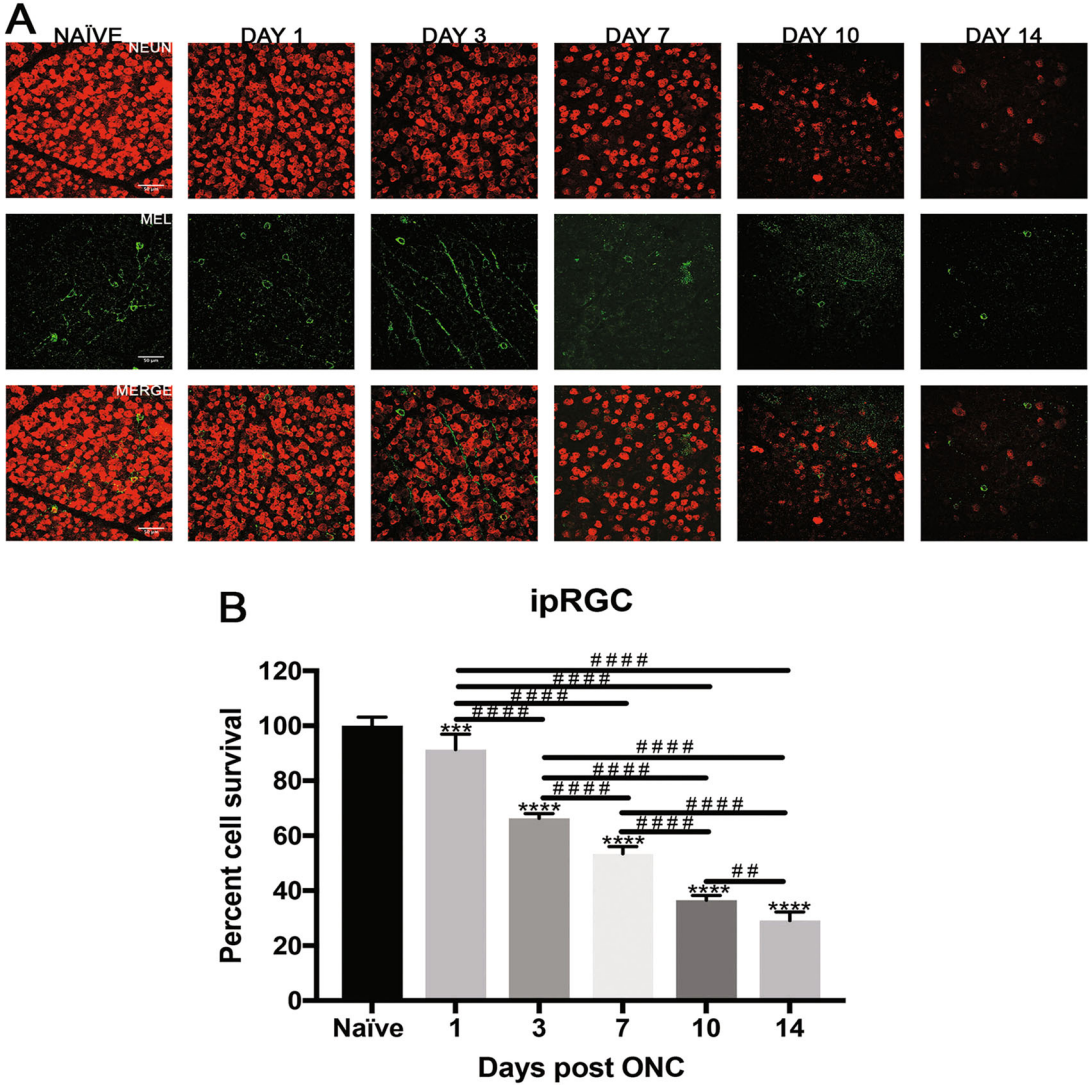
Effect of ONC injury on ipRGCs. **a** Representative images showing NeuN (red) and Mel (melanopsin) (green) immunolabeled retinal flat-mounts from C57BL/6 mice. Images of both ONC (1 day, 3 days, 7 days, 10 days and 14 days post injury) and naïve control eyes are shown. Scale bar = 50 μm. **b** Percent cell survival of ipRGCs. There was significant decline in cell survival through the 14-day time course, normalized to naïve. The values are represented as mean ± SD (*n* = 7). (* comparison to naïve, ^#^ comparison between time points). *** *p* = 0.0002, ****, ^# # # #^ *p* < 0.0001, ^# #^ *p* = 0.001, by One Way-ANOVA and Tukey’s post hoc test

**Fig. 6:**
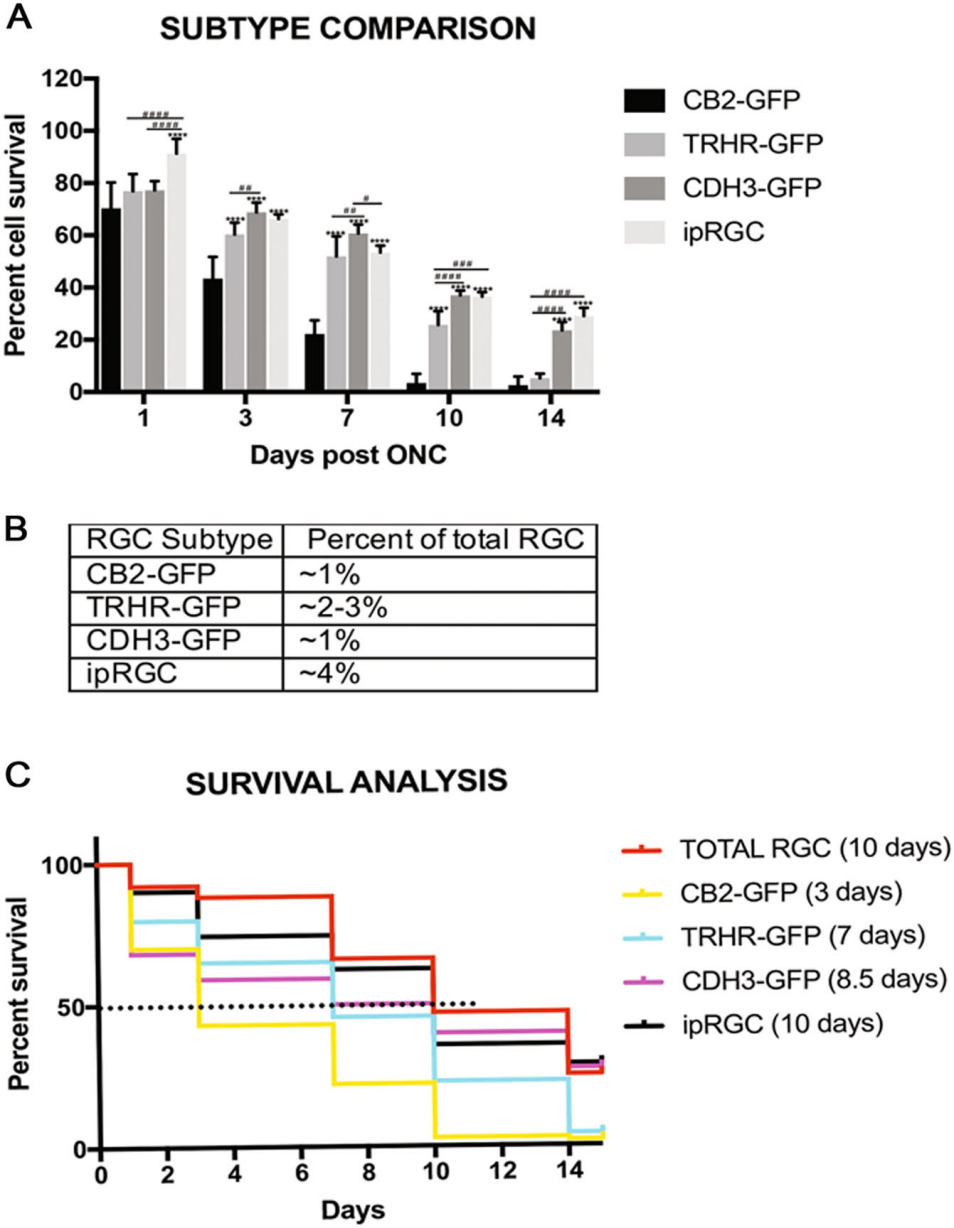
Comparison of RGC survival between strains. **a** There are varying degrees of differences in cell survival between subtypes within each time point. The values are represented as mean ± SD (*n* = 4–7). (* Comparison with CB2-GFP strain at each time point, ^#^ comparison between strains at each time point). ****, ^# # # #^ *p* < 0.0001, ^# # #^ *p* = 0.0002, ^# #^ *p* = 0.002, ^#^ *p* = 0.01 by Two Way-ANOVA and Tukey’s post hoc test. **b** Percent population of RGC subtypes out of the total RGC population in a mouse retina. **c** Survival curves of total as well as subtype specific RGCs show differences in median survival (Day post crush where 50% of the cells survive) marked by dotted line at *Y* = 50 by Kaplan–Meier plot

We utilized the ONC model to induce optic nerve injury in three transgenic mouse strains expressing GFP in three different subtypes of RGCs. We also used immunostaining of ipRGCs to assess ONC cell loss in a fourth subtype of RGC. We immunostained retinal flat-mounts to assess total as well as strain specific RGC loss in these animals. For each strain, the eyes were harvested at 1, 3, 7, 10, and 14 days post crush and compared to their respective uninjured naïve eyes. As expected, significant RGC death occurred over the 14-day time course (Fig. 1). However, the total RGC death did not vary significantly between the mouse strains at any time point. There was no difference in the temporal pattern of total RGC loss between each strain. This indicates that the ONC injury was uniform and consistent between each strain throughout the time points (Fig. 1). In contrast, we do find significant differences in the pattern of cell death in each of the specific subtypes of RGCs after optic nerve crush (Figs. 2–6).

### Alpha RGCs exhibit high vulnerability to ONC injury

CB2-RGCs are a type of alpha RGCs with large spherical soma and transient OFF physiological responses. Figure 2a shows representative images of total RGCs labeled with NeuN and CB2-RGCs labeled with GFP at each experimental time point. All time points exhibit a progressive significant loss of CB2-RGCs throughout the time course compared to naïve eyes (Fig. 2b). At one-day post-ONC, CB2-RGCs were reduced to 70.3 ± 9.9% compared to naïve (*p* < 0.0001, *N* = 4). CB2-RGCs reduced further to 43.4 ± 8.3% 3 days post-ONC (*p* < 0.0001, *N* = 5), 22.2 ± 5.2% 7 days post-ONC (*p* < 0.0001, *N* = 5), 3.5 ± 3.4% 10 days post-ONC (*p* < 0.0001, *N* = 5), and 2.6 ± 3.3% 14 days post-ONC (*p* < 0.0001, *N* = 4) compared to naïve.

### Direction selective ganglion cells undergo intermediate cell death after ONC

TRHR-RGCs are direction selective ganglion cells that project to the dorsal and ventral lateral geniculate nucleus, the superior colliculus, and the zona incerta. They are On–Off type RGCs excited by posterior motion. Figure 3a shows representative images of total RGCs labeled with NeuN and TRHR-RGCs labeled with GFP at each experimental time point. TRHR-RGCs also die rapidly post nerve injury through the 14-day time course compared to naïve eyes (Fig. 3b). One-day post-ONC, TRHR-RGCs were reduced to 76.9 ± 6.4% (*p* < 0.0001, *N* = 7), 60.3 ± 4.5% 3 days post-ONC (*p* < 0.0001, *N* = 7), 51.9 ± 7.7% 7 days post-ONC (*p* < 0.0001, *N* = 7), and 25.7 ± 5.1% 10 days post-ONC (*p* < 0.0001, *N* = 7), and 5.3 ± 1.6% 14 days post-ONC (*p* < 0.0001, *N* = 7) compared to naïve eyes.

### Non-image forming subtype cells are more resistant to nerve injury

CDH3-RGCs do not project to primary visual centers of the brain, but instead contribute to non-image forming functions. Figure 4a shows representative images of total RGCs labeled with NeuN and CDH3-RGCs labeled with GFP at each experimental time point. CDH3-RGCs demonstrate progressive cell death throughout time course compared to naïve eyes (Fig. 4b). One-day post-ONC, CDH3-RGCs reduced to 77.2 ± 3.5% (*p* < 0.0001, *N* = 7), 68.8 ± 3.7 3 days post-ONC (*p* < 0.0001, *N* = 7), 60.7 ± 3.4% 7 days post-ONC (*p* < 0.0001, *N* = 7), 37 ± 1.7% 10 days post-ONC (*p* < 0.0001, *N* = 7), and 23.6 ± 3.1% 14 days post-ONC (*p* < 0.0001, *N* = 7) compared to naïve.

ipRGCs are intrinsically photosensitive RGCs due to the presence of the pigment melanopsin. They are responsible for pupillary reflexes and other non-vision related functions. Figure 5a shows representative images of total RGCs labeled with NeuN and ipRGCs labeled with melanopsin at each experimental time point. These cells were the more resistant to injury throughout the time course when compared to naïve eyes (Fig. 5b). By day 1 post-ONC, ipRGCs decreased to 91.3 ± 5.6% (*p* < 0.0002, *N* = 7), 66.3 ± 1.6 3 days post-ONC (*p* < 0.0001, *N* = 7), 53.4 ± 2.5% 7 days post-ONC (*p* < 0.0001, *N* = 7), 36.5 ± 1.6% 10 days post-ONC (*p* < 0.0001, *N* = 7), and 29.1 ± 3% 14 days post-ONC (*p* < 0.0001, *N* = 7).

In order to evaluate the timing and onset of cell death between the RGC subtypes, we compared the cell death of each subtype at each experimental time point (Fig. 6a). Each subtype of RGC represents a small percentage of the total RGC population (Fig. 6b), but the susceptibility and resistance of each of the subtypes significantly differed from one another. The median survival for each subtype was calculated by Kaplan–Meier survival analysis, which calculates the time at which only 50% of the cells survive (Fig. 6c). CB2-RGCs have the lowest median survival of 3 days, TRHR-RGCs have a median survival of 7 days, CDH3-RGCs have a median survival of 8.5 days, while ipRGCs and total RGCs both have a median survival of 10 days. These data suggest that RGC subtypes die at different rates of post-ONC and that α-RGCs are the most susceptible and ipRGCs the most resistant in this experimental model of acute optic nerve injury.

### Axonal degeneration is independent of the subtype of RGCs

**Fig. 7:**
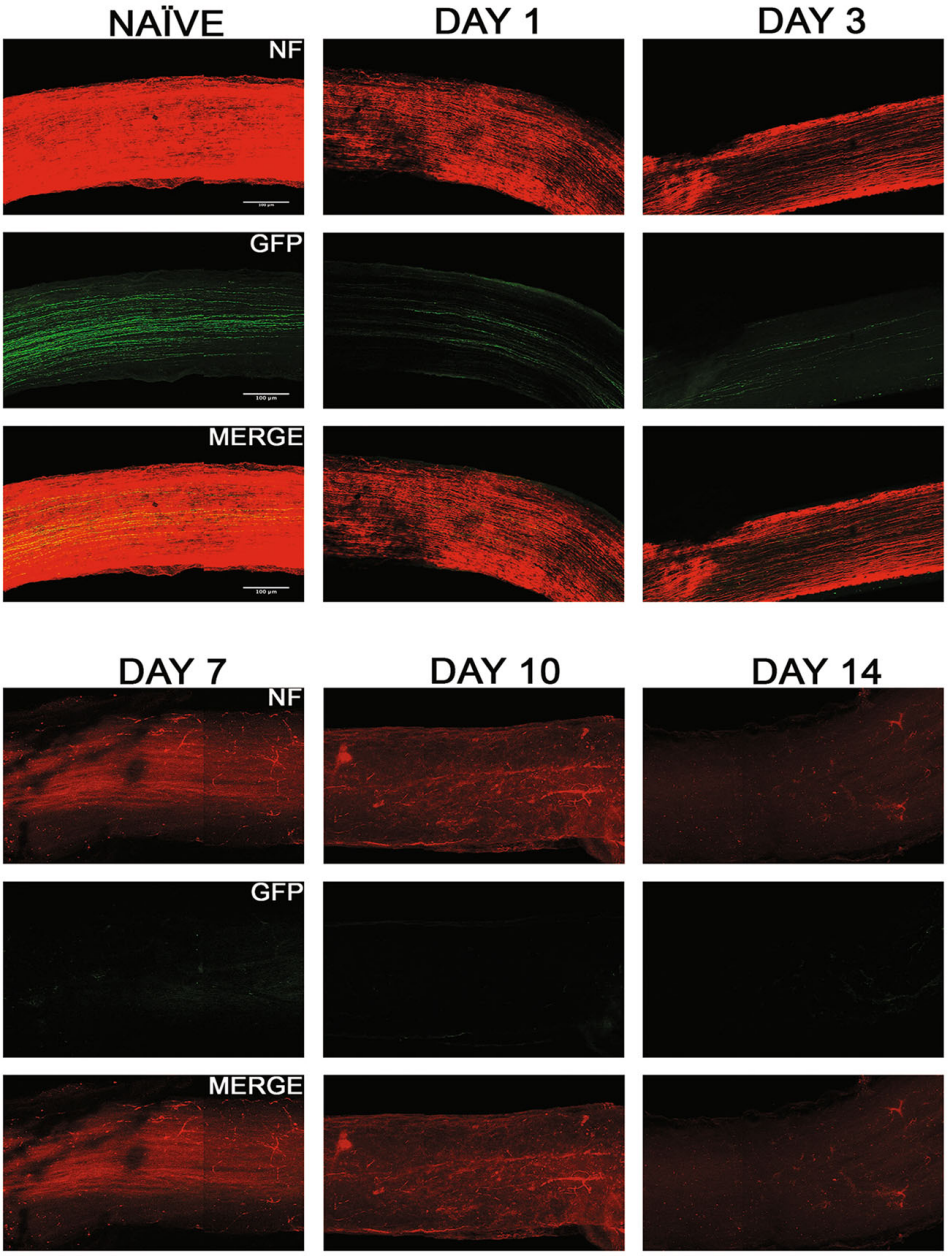
Axonal degeneration after ONC in TRHR-RGC neurons. Representative 3D images of tissue cleared optic nerve showing NF (neurofilament) (red) and GFP (green) immunolabeled whole optic nerves from TRHR-GFP mice. Images of ONC (1 day, 3 days, 7 days, 10 days and 14 days post injury) and naïve control nerves are shown (*n* = 7). Scale bar = 100 μm

**Fig. 8:**
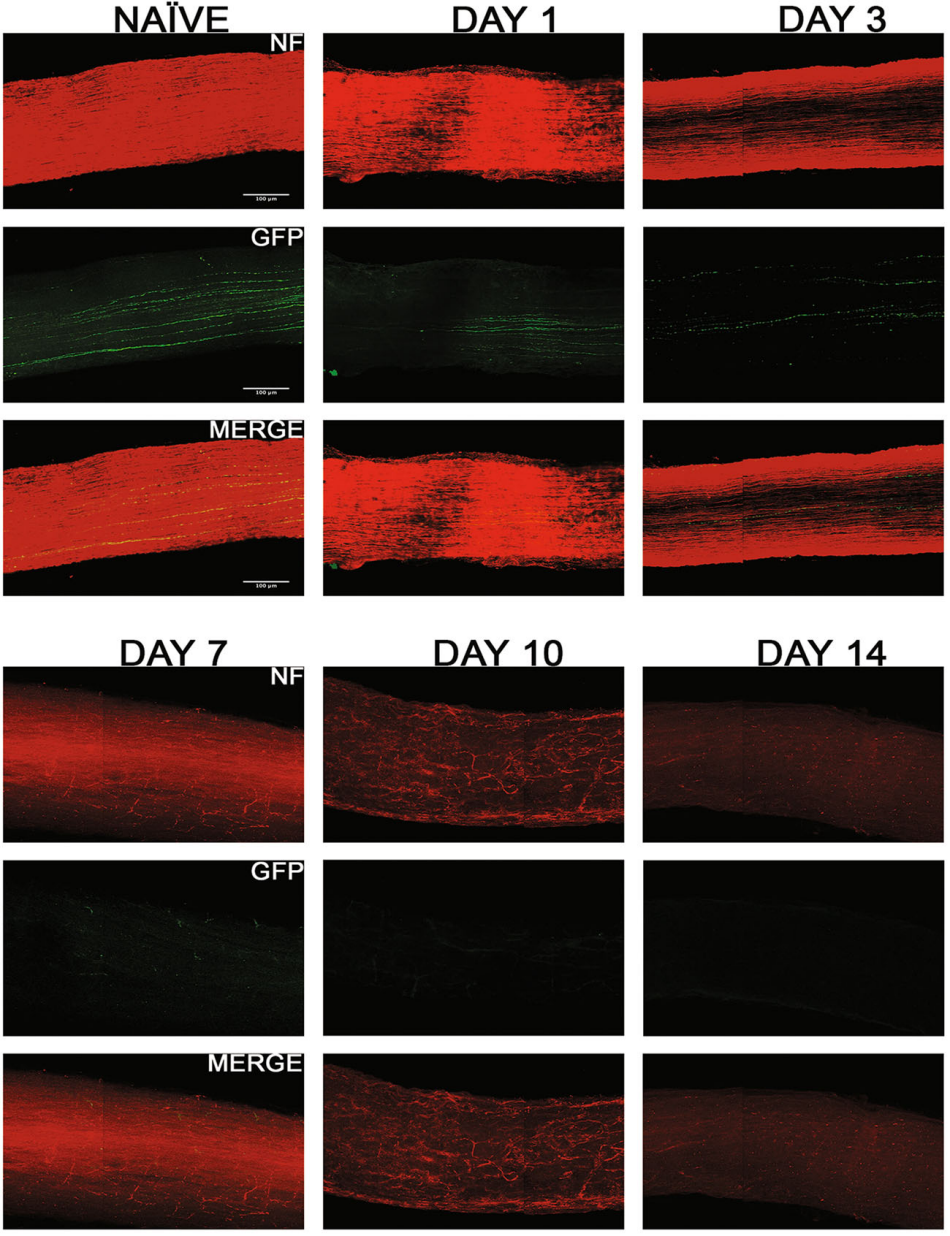
Axonal degeneration after ONC in CDH3-RGC neurons. Representative 3D images of tissue cleared optic nerve showing NF (neurofilament) (red) and GFP (green) immunolabeled whole optic nerves from CDH3-GFP mice. Images of ONC (1 day, 3 days, 7 days, 10 days and 14 days post injury) and naïve control nerves are shown (*n* = 7). Scale bar = 100 μm

**Fig. 9:**
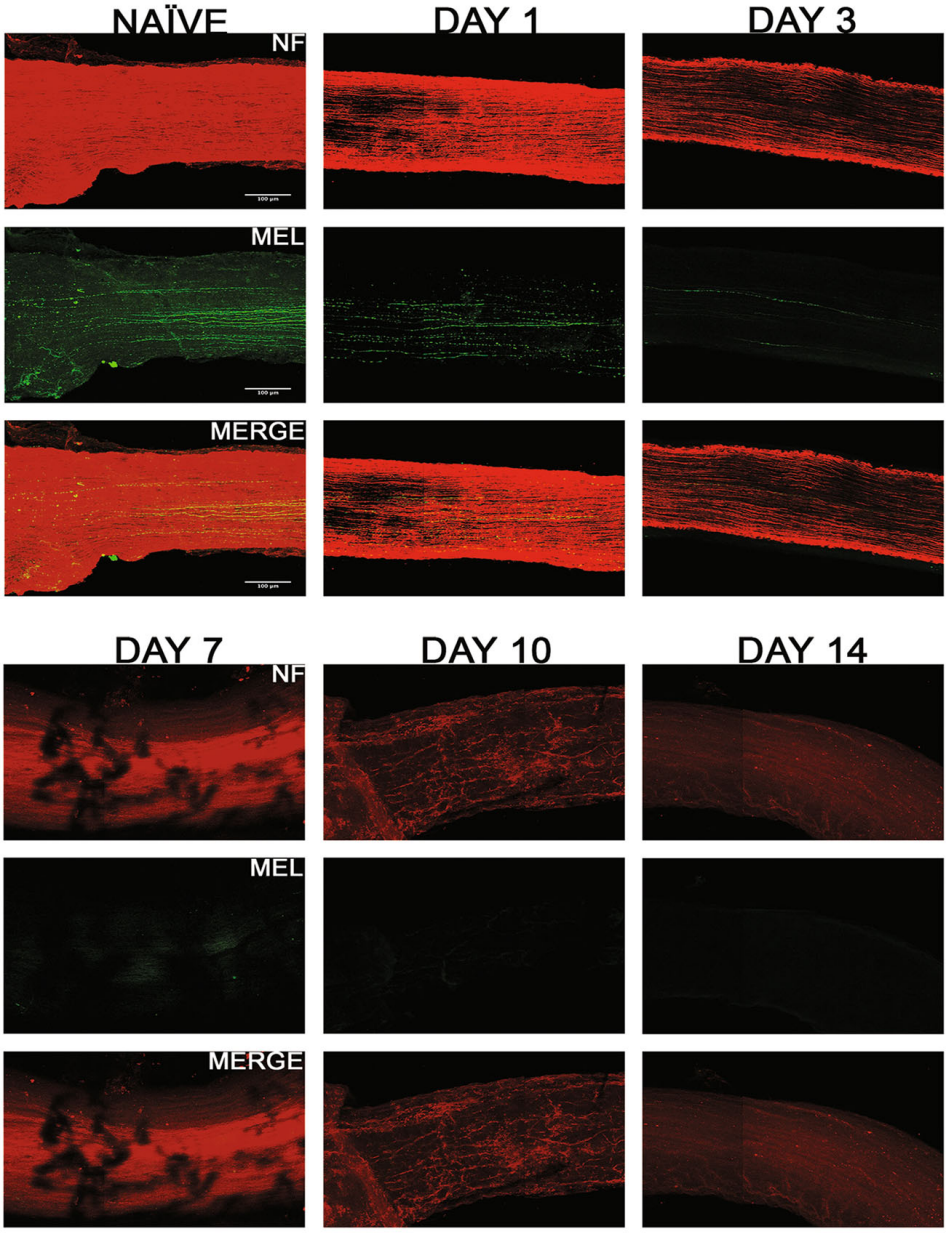
Axonal degeneration after ONC in ipRGC neurons. Representative 3D images of tissue cleared optic nerve showing NF (neurofilament) (red) and MEL (melanopsin) (green) immunolabeled whole optic nerves from C57BL/6 mice. Images of ONC (1 day, 3 days, 7 days, 10 days and 14 days post injury) and naïve control nerves are shown(*n* = 7). Scale bar = 100 μm

**Fig. 10:**
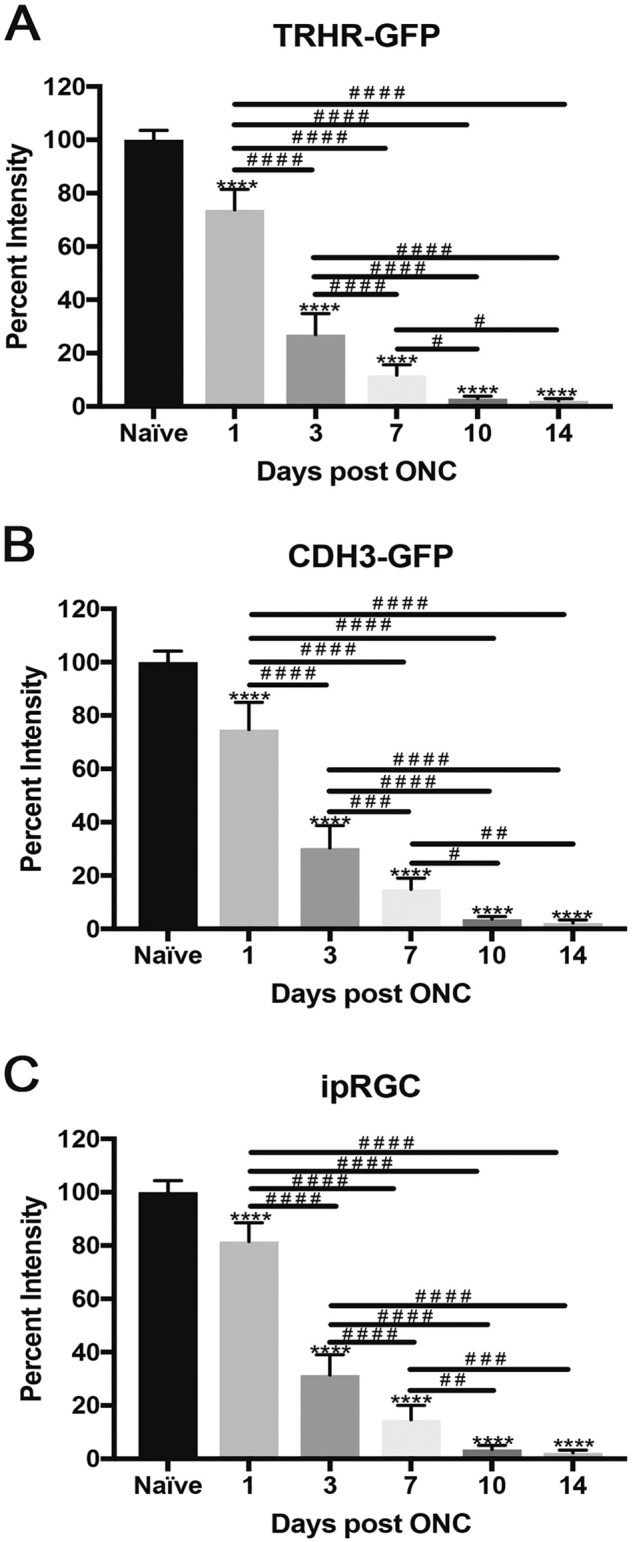
Comparison of axonal degeneration in RGC subtypes post ONC. There was significant decline in fluorescent intensity of axons of each subtype through the 14-day time course, normalized to naïve. The values are represented as mean ± SD (*n* = 7). (* Comparison with naïve, ^#^ comparison between time points). **a** Percent intensity of TRHR-RGC axons post ONC. ****, ^# # # #^ *p* < 0.0001, ^#^ *p* = 0.02, by One Way-ANOVA and Tukey’s post hoc test. **b** Percent intensity of CDH3-RGC axons post ONC. ****, ^# # # #^ *p* < 0.0001, ^# #^ *p* = 0.004, ^#^ *p* = 0.01 by One Way-ANOVA and Tukey’s post hoc test. **c** Percent intensity of ipRGC axons post ONC. ****, ^# # # #^ *p* < 0.0001, ^# # #^ *p* = 0.007, ^# #^ *p* = 0.003 by One Way-ANOVA and Tukey’s post hoc test

**Fig. 11:**
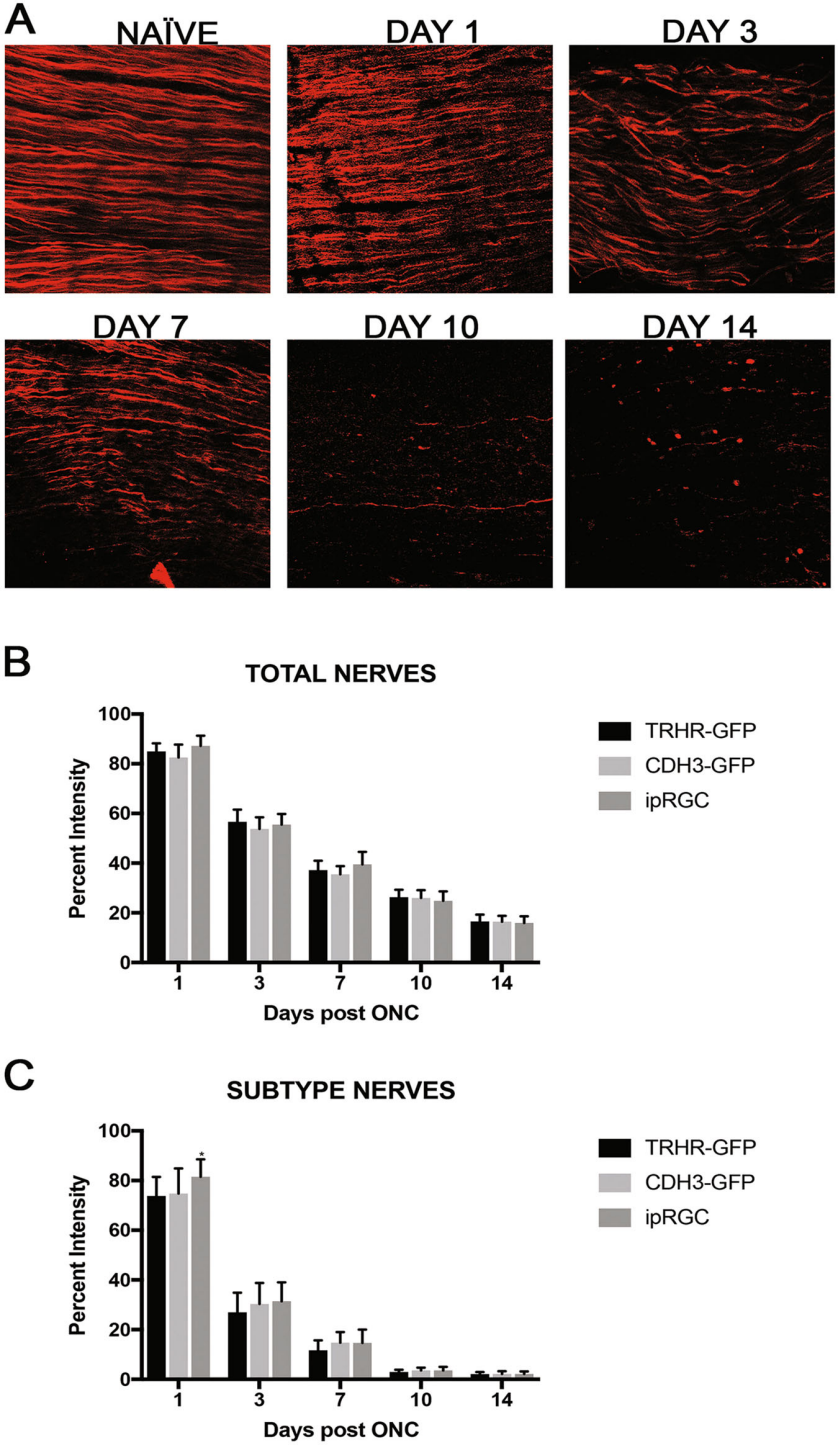
Comparison of axonal degeneration between strains. **a** Representative single plane images of optic axons labeled with neurofilament (NF) showing axons undergoing Wallerian degeneration after ONC injury. The uninjured naïve optic nerve axons have fiber-like appearance whereas the axons post ONC injury degenerate into beads on a string as prominently evident in day 10 and day 14 images. **b** Percent intensity of total RGCs between each strain within each time point shows no significant differences, by Two Way-ANOVA and Tukey’s post hoc test. **c** There are also no differences in intensities between subtypes within each time point except at day 1. The values are represented as mean ± SD (*n* = 4–7). (* Comparison with TRHR-GFP strain) * *p* = 0.02, by Two Way-ANOVA and Tukey’s post hoc test

We evaluated the axonal degeneration in each of the GFP labeled specific RGC subtype, post-ONC by immunostaining the optic nerves and measuring their intensities after 3D reconstruction (Figs. 7–9). Within each strain, there is significant decrease in intensities throughout the 14-day time course when compared to their respective naïve optic nerves: TRHR-RGCs nerves (day 1, 73.7 ± 7.6% [*p* < 0.0001, *N* = 7]; day 3, 26.9 ± 7.9% [*p* < 0.0001, *N* = 7]; day 7, 11.7 ± 3.9% [*p* < 0.0001, *N* = 7]; day 10, 2.9 ± 0.9% [*p* < 0.0001, *N* = 7]; day 14, 2.1 ± 0.8% [*p* < 0.0001, *N* = 7]) (Fig. 10a). CDH3-RGCs nerves (day 1, 74.7 ± 10.1% [*p* < 0.0001, *N* = 7]; day 3, 30.3 ± 8.3% [*p* < 0.0001, *N* = 7]; day 7, 14.8 ± 4.2%; day 10 [*p* < 0.0001, *N* = 7], 3.6 ± 1.0% [*p* < 0.0001, *N* = 7]; day 14, 2.2 ± 1.1% [*p* < 0.0001, *N* = 7]) (Fig. 10b). ipRGCs nerves (day 1, 81.5 ± 7.0% [*p* < 0.0001, *N* = 7]; day 3, 31.4 ± 7.5% [*p* < 0.0001, *N* = 7]; day 7, 14.6 ± 5.3% [*p* < 0.0001, *N* = 7]; day 10, 3.5 ± 1.4% [*p* < 0.0001, *N* = 7]; day 14, 2.2 ± 1.0% [*p* < 0.0001, *N* = 7]) (Fig. 10c). After the initial crush insult, the axons started degenerating distal to the site of injury and eventually degraded into the characteristic beads on a string formation seen during Wallerian degeneration (Fig. 11a). As expected, the total axonal degeneration was consistent between the strains at any given time point (Fig. 11b). Interestingly, even though RGC death was subtype dependent, there was no significant differences in axonal degeneration between subtypes at any time point (except day 1 TRHR-GFP compared to ipRGC, *p* = 0.029).

**Fig. 12:**
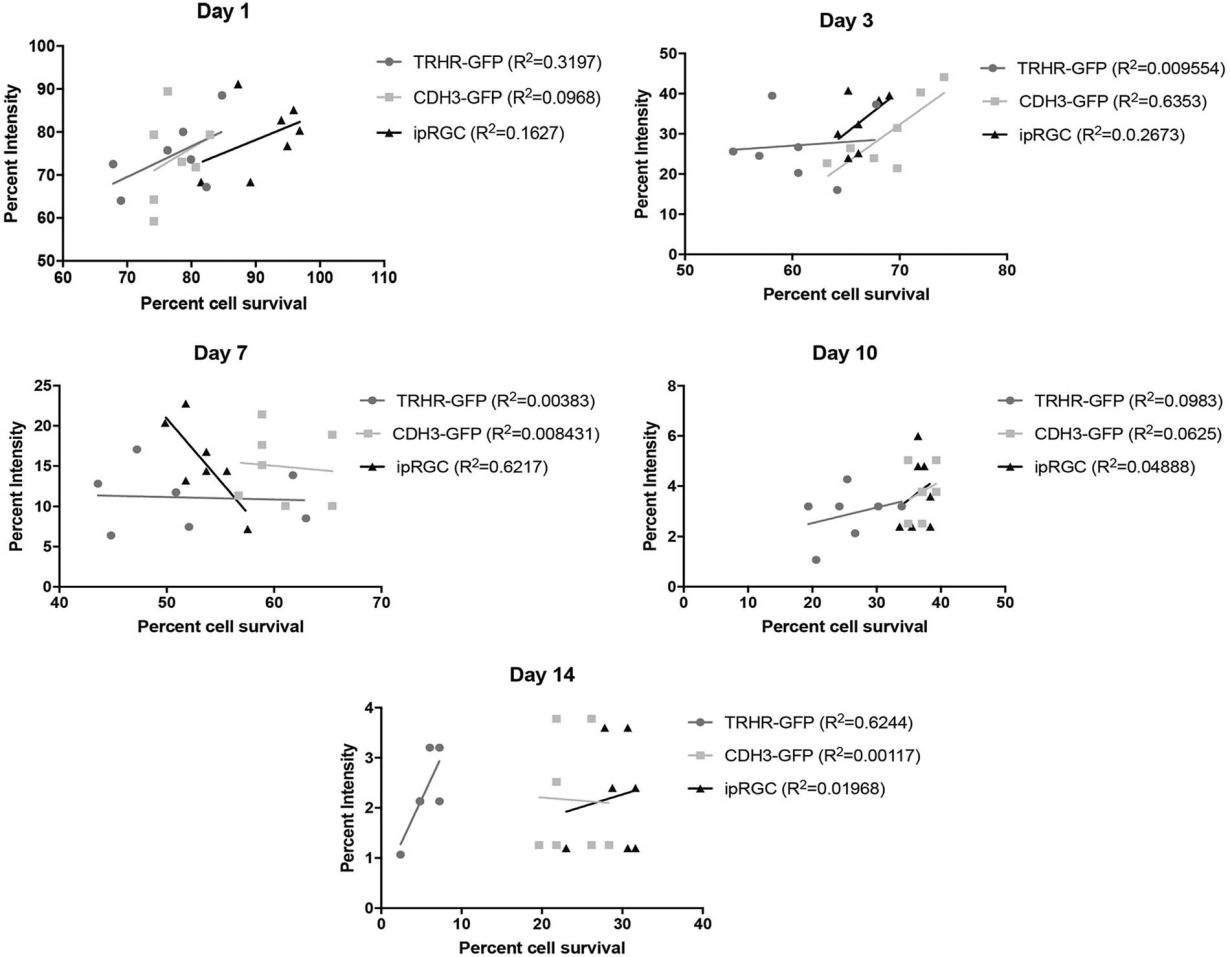
Correlation of cell survival to axonal degeneration. Scatter plots at 1 day, 3 days, 7 days, 10 days, and 14 days post injury. The plots show no correlation between cell survival and axonal degeneration of RGC subtypes at each time point. Lines per graph represent linear regression with their respective *R*^2^ values

Scatter plots of cell survival versus axonal intensity also show no correlation for any of the three subtypes at each time point (Fig. 12): day 1 TRHR-GFP *R*^2^ = 0.3197, CDH3-GFP *R*^2^ = 0.0968, ipRGC *R*^2^ = 0.1627; day 3 TRHR-GFP *R*^2^ = 0.009554, CDH3-GFP *R*^2^ = 0.6353, ipRGC *R*^2^ = 0.1627; day 7 TRHR-GFP *R*^2^ = 0.00383, CDH3-GFP *R*^2^ = 0.008431, ipRGC *R*^2^ = 0.6217; day 10 TRHR-GFP *R*^2^ = 0.0983, CDH3-GFP *R*^2^ = 0.0625, ipRGC *R*^2^ = 0.04888; day 14 TRHR-GFP *R*^2^ = 0.6244, CDH3-GFP *R*^2^ = 0.00117, ipRGC *R*^2^ = 0.01968. These data suggest that there is no correlation between progression RGC soma death and their respective axonal degeneration after ONC injury.

## Discussion

The total ganglion cell population develops from a common precursor into several different subtypes of RGCs with different physiological and visual characteristics^26,27^. During glaucomatous injury, axonal degeneration and RGC death occurs leading to loss of vision and irreversible blindness^1,6^. Many previous studies have considered RGC subtypes to have different susceptibility to glaucomatous injury^28–33^. In our study, we show that the 4 RGC subtypes we evaluated have different cell death sensitivities to ONC injury. The alpha RGCs are the most susceptible to ONC injury followed by DSGC RGCs. The non-image forming RGCs were less susceptible to injury, with ipRGCs being the most resistant to injury of all four subtypes studied. We also observed progressive axonal degeneration in the injured nerves after ONC injury. Unlike the specific response of RGC subtype soma, we do not observe any differences in specific RGC subtype axons to injury. On the contrary, our study shows that once the nerve injury is initiated, axons start degenerating independent of their respective RGC soma. Although we used GFP and melanopsin expression to quantify death RGC subtype, previous data suggests decrease in expression of these markers precedes cell death^34^, therefore our timeline for cell death estimation may be sooner than the actual timeline. That being said, our method for quantification was uniform throughout and so we anticipate that our data regarding the pattern and differences in cell death between the RGC subtypes would remain the same.

### RGC subtype vulnerability and its implications

Retinal ganglion cells are the output neurons of the retina that relay information to the visual centers of the brain. Progressive RGC loss and axonal degeneration in glaucoma leads to the loss of connection between the eye and the brain, which leads to loss of vision. There are only about 50,000 RGCs per mouse retina that are responsible for receiving and transmitting all the complex visual information to the brain^35^. To do this more efficiently, these RGCs are divided into different subtypes that are responsive to different stimulations, have different afferent and efferent synaptic connections, physiological properties, and molecular signatures^7^. Previous studies have shown that the functional and morphological degeneration of RGCs in various experimental models of glaucoma are subtype-dependent^28^. It has been shown that functional responses and receptive fields of OFF cells were impaired earlier than ON cells after ONC injury^29^. In another study using a laser-induced mouse model of chronic ocular hypertension, it was shown that mono-laminated ON cells were more susceptible to IOP elevation than bi-laminated ON-OFF cells^30^. A study using a microbead model for IOP elevation in mice showed that OFF-transient RGCs exhibited a more rapid decline in both structural and functional organizations, the light-evoked responses of OFF-sustained RGCs were disturbed, and the ON-transient and ON-sustained RGCs had reduced spontaneous and light-evoked firing rates^31^. Another study using microbead induced IOP elevation showed that the RGCs stratifying most of their dendrites in the Off sublamina were the first to undergo structural alterations^32^. Previous work has also demonstrated that ipRGCs are more resilient to ONC as well as ocular hypertension injuries^33^. Our study corroborates the findings of these other studies that transient-Off alpha RGCs are more susceptible to injury and that ipRGCs are more resilient, as well as sheds light on cell death response of two other RGC subtypes. All these studies clearly imply that RGCs respond in a subtype specific manner to optic nerve and glaucomatous injury. It will be important to discover and describe all the RGC subtypes and their specific responses to glaucoma to better understand the progression of the disease.

### Cell-independent degeneration of axons after injury

Conventionally it has been thought that neuronal axons are highly dependent on their cell soma for survival and degenerative cues and that axonal degeneration is a passive process. However, recent studies show that axons have autonomous mechanisms independent of their cell bodies that dictate their degeneration known as Wallerian degeneration^36–38^. Wallerian degeneration occurs when a nerve fiber is injured and the part of the axon distal to the injury degenerates and degrades into beads on a string-like formation^39^. There are many studies suggesting that axonal degeneration and somal apoptosis differ in their mechanistic pathways^40–42^. Although a few regulators of Wallerian degeneration such as NMNAT (nicotinamide mononucleotide adenylyltransferase) in the *Wld*^s^ mice (mice having delayed Wallerian degeneration) as well as SARM1 (sterile alpha and TIR motif containing 1) and PHR1 (phosphate starvation response 1) have been reported, the molecular pathway of Wallerian degeneration is still unclear^38^. Wallerian degeneration in the central nervous system (CNS) is slower than in the peripheral nervous system (PNS) as oligodendrocytes (myelinating cells of CNS) lack the ability to clear debris compared to Schwann cells (myelinating cells of PNS)^43, 44^. As a result, we find granular debris of axons present long after the degeneration of the axon. In our study, we also find that the axons degenerate in a cell-independent manner and granular debris is seen throughout the nerve days after axonal degeneration.

### Glaucoma: diagnostic, neuroprotective and regenerative strategies

As glaucoma progresses slowly, the visual changes associated with it are very gradual and often go undetected until later stages of disease. Currently the diagnostic tools used to detect glaucoma and its risk factors are tonometry, ophthalmoscopic exams, visual field tests as well as electrophysiological evaluations. Out of these tests, electrophysiological evaluations are one of the more promising tools to detect early changes in glaucoma. However, the current electrophysiological exams (pattern electroretinography, multifocal electroretinography and multifocal visual evoked potentials) are limited in their potential for early detection. This limitation can be overcome by our knowledge of RGC subtype susceptibility to glaucoma and their electrophysiological properties. This information can be used to generate electrophysiological tests that are designed to detect the changes in the more susceptible subtypes of RGCs, and in doing so will detect early changes that occur during onset of glaucoma.

Studies using experimental models of glaucoma have demonstrated that there are many molecular signals and pathways that ensue once glaucomatous injury is initiated including axonal transport failure, neurotropins and neurotrophic factor deprivation, activation of intrinsic and extrinsic apoptotic signals, mitochondrial dysfunction, excitotoxic damage, oxidative stress, pathogenic reactive glia and loss of synaptic connectivity^45^. Pro- and anti-apoptotic pathways play an important role in RGC survival in glaucoma including MAP-kinase pathway, PI-3 kinase/Akt pathway, Bcl-2 family, caspases, and IAP family, and by utilizing these pathways some degree of RGC protection has been shown in many studies^46–51^. In addition, neurotrophic factors and neurotrophins have been used for RGC and axon survival^52–55^. Mitochondrial dysfunction as well as oxidative stress has been shown to contribute to RGC loss and axonal degeneration during injury^56–60^. The immune system also contributes to pathophysiology of glaucoma both in the retina and the optic nerve. In the retina reactive gliosis and complement activation both lead to progressive RGC loss^61–64^, whereas slow phagocytic activity of microglia in the optic nerves leads to accumulation of degenerating myelin and glial scarring^65,66^.

As demonstrated in our current study, axonal degeneration and RGC death appear to follow different pathways after ONC injury and even though these pathways may have some overlapping contributors, utilization of a single approach will not preserve both RGCs and axons. Preserving both RGC somas and axons is necessary for true neuroprotection. Also, some approaches for RGC preservation are detrimental to axonal regeneration such as inhibition of phagocytic and complement activity^63,67,68^. Therefore, a true therapeutic strategy for glaucoma would involve strategies for neuroprotection as well as neuro-regeneration while taking into consideration the dynamic molecular and physiological nature of ganglion cell neurons.

## Materials and methods

### Mice

Breeding pairs of mouse strains were obtained from Mutant Mouse Regional Resource Centers namely, Tg(Calb2-EGFP)CM104Gsat/Mmmh (RRID:MMRRC_000283-MU) hereafter referred to as CB2-GFP, B6;FVB-Tg(Trhr-EGFP)HU193Gsat/Mmucd (RRID:MMRRC_030036-UCD) hereafter referred to as TRHR-GFP, and Tg(Cdh3-EGFP)BK102Gsat/Mmnc (RRID:MMRRC_000236-UNC) hereafter referred to as CDH3-GFP. TRHR-GFP and CDH3-GFP mice were backcrossed to the C57BL/6J background (Jackson Laboratory, Bar Harbor, ME) for more than 10 generations in our laboratory. C57BL/6J animals were also used to study ipRGCs. There was a loss of GFP transgene expression during backcrossing of CB2-GFP animals to the C57BL/6J background, hence these animals could only be used on their original Swiss Webster background. In addition, due to these breeding difficulties we were only able to analyze the RGCs in the CB2-GFP strain and did not have the ability to analyze the optic nerves in this strain. All mice were maintained in 12:12 light/dark cycle and supplied with food and water ad libitum. All mice used in these experiments were between 1–3 months of age. Both male and female mice were used for these studies. All experiments were conducted in accordance with the ARVO Statement of the Use of Animals in Ophthalmic and Vision Research and the University of North Texas Health Science Center’s Institutional Animal Care and Use Committee guidelines.

### Optic nerve crush (ONC)

Mice (*N* = 5–7/strain) were subjected to ONC using the Nickells technique^18^. For the crush surgery, mice were anesthetized by intraperitoneal (ip) injection of Ketamine/Xylazine (100/10 mg/kg) and ONC was performed intraorbitally. In right eye of each mouse, the conjunctiva was cut in the lateral canthus region and gently peeled back. The optic nerve was then exposed through a small window made between the surrounding muscle and approximately 0.5 mm posterior to the globe the optic nerve was crushed for 4 s using a self-closing jeweler’s forceps. Care was taken not to damage muscles or blood vessels causing retinal ischemia. Naïve (uncrushed) animals from each strain were used as their respective controls.

### Tissue harvest (optic nerve and retina)

Mice were sacrificed at 0, 1, 3, 7, 10 and 14 days post crush by deeply anesthetizing them with a mixture of Ketamine/Xylazine/Acepromazine (100/10/3 mg/kg) (ip). The animals were then transcardially perfused with phosphate buffer saline (PBS) (pH 7.4) followed by 4% paraformaldehyde (PFA) in PBS. Eyes along with the optic nerves were dissected and post fixed for 2 h in 4% PFA at room temperature, then rinsed in PBS. Following fixation, retinas and optic nerves from fixed eyes were carefully dissected and processed for cell count and axonal analysis.

### Retinal flat mount

Dissected retinas were pre-treated in 0.3% TritonX-100 in PBS for 30 min (x4) and then blocked in 0.3 % Triton X-100 in PBS containing 10% goat serum for 2 h. Subtype-specific RGCs were labeled using anti-GFP (1∶1000; #A6455, Molecular Probes, Eugene, OR, USA) or anti-Melanopsin (1∶1000; #ABN38, ATSbios, San Diego, CA, USA) antibodies and total RGCs were labeled using anti-NeuN (1:1000; Chemicon) antibody overnight at 4 °C. Following washes in PBS, the retinas were incubated with AlexaFluor488 goat-anti-rabbit (1:1000, diluted in PBST) and AlexaFluor594 goat-anti-mouse (1:1000, diluted in PBST) overnight at 4 °C and mounted with Vectashield Mounting Medium containing DAPI (Vector Laboratories). Eight (40x zoom 0.7, 0.09 mm^2^) images were taken from peripheral and central regions of the four quadrants of each retina using LSM 510 Zeiss confocal microscope. Cells were counted both manually and by using imageJ (FIJI) Cell Counter Plugin (Supp.Fig.1). Briefly, a.Tiff format of the image to be analyzed was loaded into imageJ. The file was converted to an 8-bit image and run through FTT bandpass filter. The image was then run through auto-threshold and converted to a binary image. After removing outliers, binary functions like Fill holes and Watershed were applied. This created a binary image. By applying appropriate particle parameters (size, circularity) the Analyze particle command was run which gave the output of cell outline and cell count. For each individual retina, the RGC count was obtained by averaging the eight counts for each retina.

### Optical clearing of optic nerve

Optic nerves were cleared using the passive clarity technique^69^. The nerves were briefly rinsed 3x with PBS for 10 min. PBS was replaced by 4% hydrogel solution (0.0025 g/ml of VA-044 activator in 4% acrylamide solution). The vial was over filled and capped to avoid any air bubbles. The nerves were then incubated at 4 °C for 6–8 h followed by 1 h incubation at room temperature. The hydrogel solution was removed, and the nerves were briefly rinsed in 10X PBS followed by three 5 min washes in 1X PBS. Sodium dodecyl sulfate (SDS) solution 8%, with 0.5% 2-mercaptoethanol was added and the nerves were incubated at 37 °C with gentle agitation. The nerves clear within 1–2 days. They were then washed four times in 0.1% sodium azide in 0.1% Tween 20/1X PBS over a span of 24 h.

### C-PRESTO (Centrifugation-pressure related efficient and stable transfer of macromolecules into organs) to immunolabel nerves

The C-PRESTO technique was used to immunolabel the optic nerves^70^. The nerves were transferred into 1.5 mL centrifuge tubes containing 500 µL each of the primary antibodies in antibody dilution solution (3% goat serum/0/1% Triton X-100/1XPBS): anti-GFP (1∶1000; #A6455, Molecular Probes, Eugene, OR, USA) or anti-Melanopsin (1∶250; #ABN38, ATSbios, San Diego, CA, USA), and anti-Neurofilament (1:200; #M0762, DAKO, Santa Clara, CA, USA). The tubes were then centrifuged at 600×*g* for 2 h followed by a wash with 0.1X PBS by centrifugation at 600×*g* for 30 min. AlexaFluor488 goat-anti-rabbit (1:1000, diluted in antibody dilution solution) and AlexaFluor594 goat-anti-mouse (1:1000, diluted in antibody dilution solution) were added and the tubes were centrifuged at 600×*g* for 2 h. The labeled samples were washed with 0.1X PBS by centrifugation at 600×*g* for 30 min. PBS was replaced with RIMS (RIMS is 40 g of histodenz in 30 ml of PB at a pH of 7.5) solution just enough to cover the tissue for overnight. The nerves were then mounted on a slide in RIMS solution with a coverslip. The nerves were imaged using Z-stacks and Tile function with maximum intensity (LSM 510 Zeiss confocal microscope). Optic nerve 3D projections were created using the ZEN software. ImageJ software was used to analyze consistent ROI (regions of interest) for each sample (Supp.Fig.2). Mean fluorescent intensity was recorded using the analysis and measure tool.

## Electronic supplementary material

Supplemental Figure 1

Supplemental Figure 2

Supplementary figure legends
