# Supplementary figures and images for: Subtype-specific response of retinal ganglion cells to optic nerve crush
Source: Cell Death Discov. 2018 Jun 28;4:67. doi: 10.1038/s41420-018-0069-y (PMC6054657; doi:10.1038/s41420-018-0069-y)

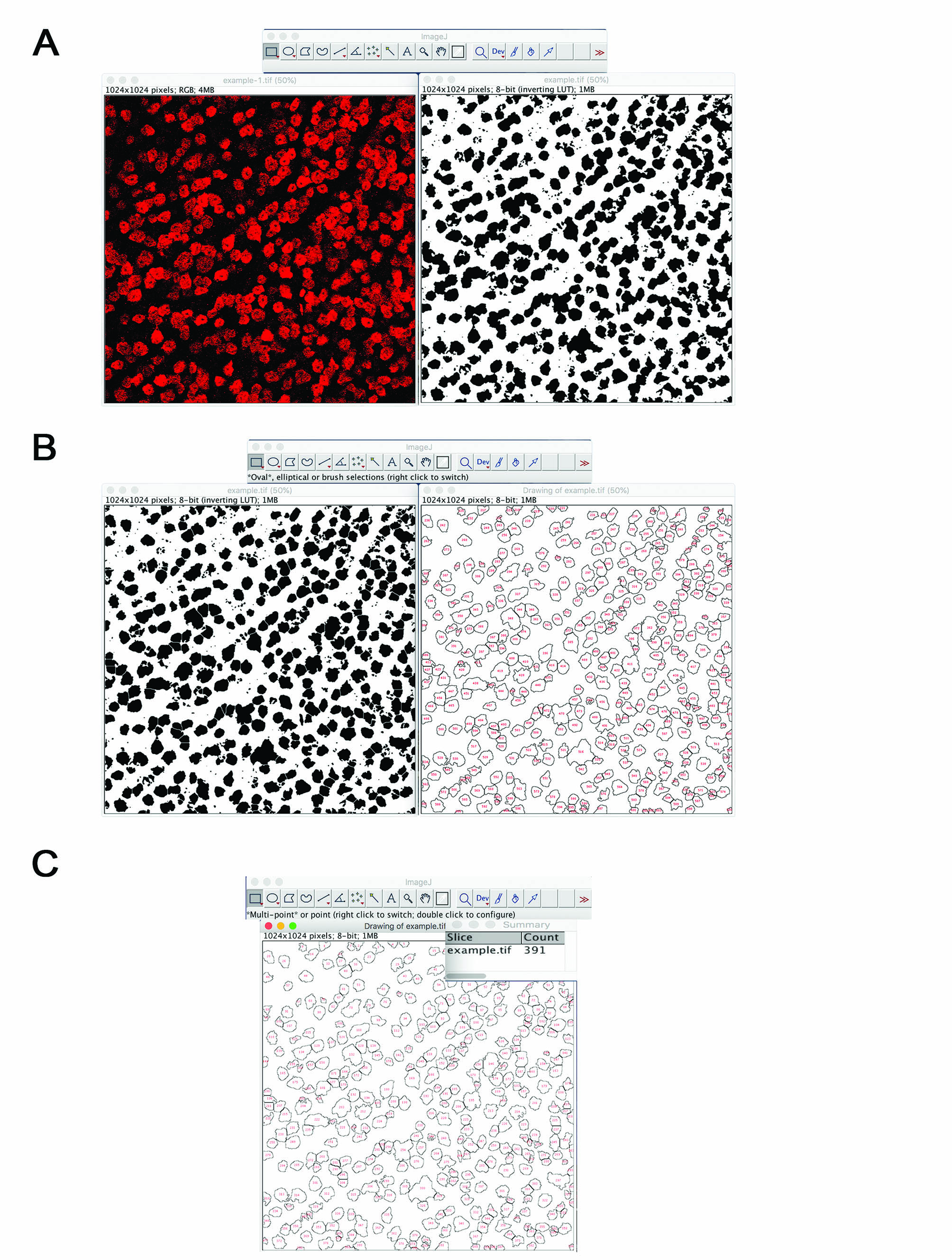

Supplement: Supplementary file 1 — Supplemental Figure 1 [file 41420_2018_69_MOESM1_ESM.jpg]

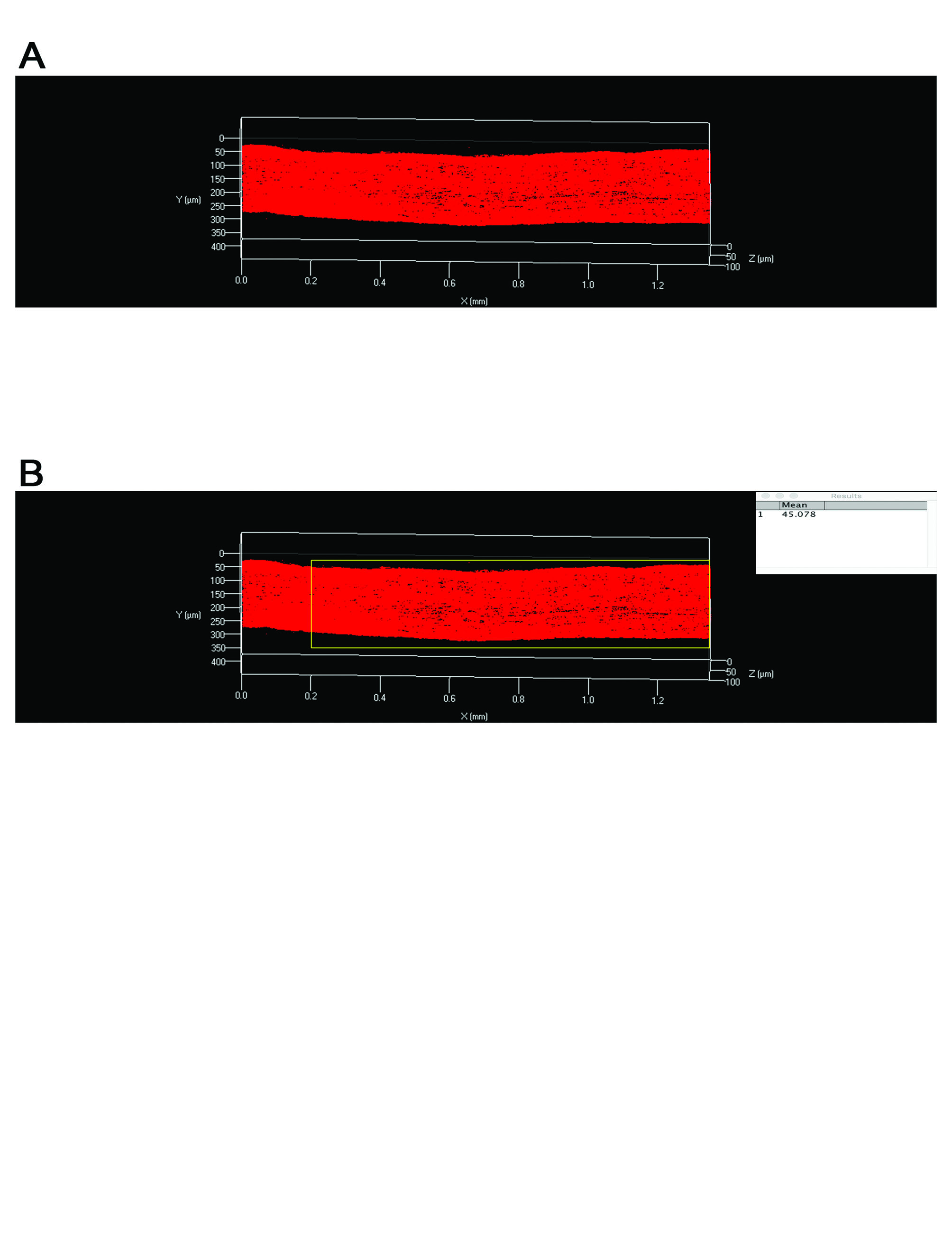

Supplement: Supplementary file 2 — Supplemental Figure 2 [file 41420_2018_69_MOESM2_ESM.jpg]
