# Supplementary material for: Subtype-specific response of retinal ganglion cells to optic nerve crush
Source: Cell Death Discov. 2018 Jun 28;4:67. doi: 10.1038/s41420-018-0069-y (PMC6054657; doi:10.1038/s41420-018-0069-y)
Supplement: Supplementary file 3 — Supplementary figure legends [file 41420_2018_69_MOESM3_ESM.docx]

**Supplemental Figure 1. RGC count using imageJ.** **(A)** Tiff image after threshold and binary processing. **(B)** Cells outlined after particle analysis. **(C)** Example of the result output as cell count after imageJ analysis.

**Supplemental Figure 2. Measuring fluorescent intensity using imageJ. (A)** 3D image of whole optic nerve with coordinates. **(B)** Intensity measurement using ROI (Region of interest) from the crush site (0.2 mm) through the whole nerve.
